# Toxicity reduction in continuous, high productivity ethanol fermentation by *Parageobacillus thermoglucosidasius* using in situ microbubble gas stripping

**DOI:** 10.1186/s12934-025-02754-5

**Published:** 2025-06-18

**Authors:** Christopher Ibenegbu, William B. Zimmerman, Michael Hines, Pratik D. Desai, H. C. Hemaka Bandulasena, David J. Leak

**Affiliations:** 1 Department of Life Sciences, University of Bath, Bath, BA2 7AY UK; 2 Department of Chemical and Biological Engineering, University of Sheffield, Sheffield, S1 3AD UK; 3 Perlemax Ltd, Kroto Innovation Centre, 318 Broad Lane, Sheffield, S3 7HQ UK; 4 Department of Chemical Engineering, University of Loughborough, Loughborough, LE11 3TU UK

## Abstract

Ethanol concentrations above 4% (v/v) are required for economic bioethanol production due to the cost of recovery from dilute solutions. Although thermophilic bacteria have many potential advantages over *Saccharomyces cerevisiae* as process organisms for second generation bioethanol production, they are known to be less tolerant to ethanol, typically to concentrations less than 4% (v/v). To address this issue we have investigated the application of in situ gas-stripping of ethanol using microbubbles to increase the surface area per unit volume of gas, using fed-batch and continuous cultures of the engineered ethanologenic thermophile *Parageobacillus thermoglucosidasius* TM242. By using microbubbles generated at room temperature using a Desai-Zimmerman Fluid Oscillator, we initially operated a mixed batch and fed-batch fermentation, followed by a continuous fermentation and finally a chemostat fermentation, under conditions which would have generated in excess of 4% (v/v) ethanol. In all cases, gas stripping maintained the actual dissolved ethanol concentration below, or close to toxic levels. As the focus of this study was on demonstrating the efficiency of in situ microbubble gas stripping, to simplify the operation the latter two processes involved a combination of produced and supplemented ethanol, with the chemostat culture producing a nominal maximum 7.1% v/v based on glucose used (5.1–5.3% (v/v) based on ethanol recovered). This offers a practical way to produce second generation bio-ethanol from thermophiles.

**Supplementary Information:**

The online version contains supplementary material available at 10.1186/s12934-025-02754-5.

## Background

Bioethanol blends with petrol are now mandatory in many countries, with 10% (v/v) mixtures (E10) for regular internal combustion engines becoming increasingly common and 85% (E85) for modified flex-fuel vehicles common in the USA. Compared to pure fossil-fuels, substitution with fermentation-derived ethanol has a beneficial effect on net greenhouse gas emissions [1], although there has been controversy over the use of starch-based feedstocks which can also be used as food. To avoid this problem, producers have been encouraged to increase the use of lignocellulose-derived feedstocks, either exclusively or in combination with the primary sugar. Examples of the latter include sugar-cane bagasse and corn-stover. However, the economics of using lignocellulose-derived feedstocks (so-called second-generation processes) are marginal, even in a fully functioning economy.

One problem with using second-generation feedstocks is that the hemicellulose component of lignocellulose is predominantly composed of pentose sugars such as xylose and arabinose, which neither wild-type *Saccharomyces cerevisiae* or *Zymomonas mobilis*, the organisms traditionally associated with bioethanol production, can naturally ferment [2–6]. While recombinant pentose-utilising strains of both *S. cerevisiae* and *Z. mobilis* have been produced [5–9], the move to more complex feedstocks with the associated requirement for extensive pre-processing raises the question of whether there are better process organisms available. For instance, after physico-chemical pretreatment, the cellulose and hemicellulose are typically converted to monomeric sugars by 48–72 h enzymatic hydrolysis at 50–55 °C. This is followed by fermentation at 30–35 °C. A thermophilic ethanol producer growing at 55–60 °C would allow integration with enzymatic hydrolysis, for example a partial simultaneous saccharification and fermentation, but would also not require cooling during fermentation (which is exothermic).

*Parageobacillus thermoglucosidasius* TM242 is a thermophilic ethanologen developed by TMO Renewables Ltd to exploit these useful properties. In addition to the ability to grow on monomeric pentoses it can transport and subsequently catabolise hemicellulose-derived oligomers and cellobiose [10–12], properties which provide a competitive advantage in its ecological niche, but which are valuable in second generation bioprocesses as this reduces the requirement for enzyme pretreatment. However, like all thermophiles characterised to date [13–15] it has a low ethanol tolerance. Both *S. cerevisiae* and *Z. mobilis* can tolerate ethanol concentrations higher than 10% (v/v) [2, 16–21]. Given the high cost of distillation which can comprise up to 30–40% of the total cost of 1st generation ethanol production [16, 22], it has been estimated that ethanol concentrations of less than 4% (v/v) would not be economic to recover [23]; a typical batch yeast fermentation is run to about 10–12% (v/v) ethanol before harvesting [2, 16]. The growth of *P. thermoglucosidasius* starts to be affected above 2% (v/v) ethanol and is completely inhibited by 4% (v/v), which is a significant “Achilles heel” [24]. However, it is clear when running ethanologenic fermentations at 60 °C that a significant amount of ethanol is stripped into the gas stream [23, 25] as a result of sparger aeration and the effect of temperature on vapour pressure. Simulation studies show that it would actually be possible to operate at commercially relevant productivities using gas-stripping of ethanol, but with traditional sparger aeration this would require unfeasible gas flow rates [25]. A critical factor determining the effectiveness of gas stripping is the gas–liquid interfacial area for mass transfer and mixing efficiency within the bubbles [26–30] so, as an alternative to increasing gas flow rates, a reduction in bubble size should have the same effect and could allow adequate stripping with moderate gas flow rates. This also follows from work done by Desai et al., [31] showcasing hot microbubble injection at low flows to strip off volatile components in a continuously operating system. To evaluate this, we have explored the use of microbubbles generated inexpensively using a Desai Zimmerman Fluidic Oscillator (DZFO) in both fed batch and chemostat cultures, which can be scaled up to industrial scale. While the materials of construction of the proprietary prototype in situ microbubble generator compromised long-term aseptic operation, using simple room-temperature gas supply we have demonstrated that gas-stripping with microbubbles is a feasible solution, which would facilitate continuous operation. Scalable sterilisable systems using this technology have been demonstrated to remain sterile in a continuously operating environment, although scaling down for lab-scale studies has been an issue. In previous studies involving gas stripping in a recirculation loop [32] we have shown that the use of high temperature microbubbles (~ 75 °C) does not compromise the viability of *P. thermoglucosidasius* but can enhance gas-stripping considerably, suggesting that continuous production of at least the equivalent of 10% (v/v) ethanol is feasible using this approach. In this study we have investigated the feasibility of direct in situ gas stripping, a technically simpler approach which avoids the requirement for rapid recirculation through an external loop. As in the previous study [32] the aim was to establish the stripping rates that could be achieved while maintaining ethanol concentrations in the bioreactor below toxic levels.

## Materials and methods

### Organism, growth media and inoculum preparation

*Parageobacillus thermoglucosidasius* strain TM242 (*ldh*^−^, *pfl*^−^, *pdh*_up_), supplied by TMO Renewables (now ReBio Ltd) was initially grown at 60 ˚C in 2SPY media (soya peptone 16 g/L, yeast extract 8 g/L and sodium chloride 5 g/L) and stored in 18% (v/v) glycerol in vials at − 80 ℃. A single vial was used to prepare overnight plate cultures by spreading 100 μL of the stock on Tryptone Soya Agar plates (TSA) composed of casein peptone (pancreatic) 15 g/L, soya peptone (papaic) 5 g/L, sodium chloride 5 g/L and agar 15 g/L. The TSA plates were incubated at 60 ℃ for 18 h. Subsequently a loopful of bacteria from the plate was used to inoculate 4 × 250 mL baffled conical flasks each containing 50 mL of 2SPY media and incubated at 60 ℃ in an orbital incubator (Innova 44 incubator shaker, New Brunswick Scientific Ltd, St Albans, UK) with shaking at 200 rpm for 4 h (OD_600_ 8–12). These 4 flasks were subsequently pooled together and used as inoculum for the bioreactors.

For both fed-batch and continuous bioreactor operation, cells were grown at 60 °C in 2SPY medium supplemented with different concentrations of glucose. Media containing glucose was filter-sterilised through a 0.2 µm MilliQ filter (Merck Life Sciences, UK). For continuous operation, freshly prepared media adjusted to pH 5 with 5M H_2_SO_4_ was pumped (i150 peristaltic pump with C1R3 pump head, iPumps Ltd, UK) from a chilled (to minimise evaporation), continuously stirred reservoir through a 0.2 µm MilliQ filter, with the pumping rate pre-calibrated to give the required flow rate. The dilution rate, D (0.1 /h for both continuous and chemostat operation) was calculated as F/V, where F was the feeding rate in mL/h, and V was the working volume (mL) in the bioreactor. The reactor working volume was controlled by a pump on the Biostat controller activated by PID control from a load sensor. Control of pH in the bioreactor was by automatic addition of 5M NaOH in response to the output from an EASYFERM PLUS VP pH/Rx 255 electrode (Hamilton, Switzerland) while sterile antifoam (antifoam 204, Sigma, UK) was added automatically based on activation of the Biostat B-plus high-foam sensor (Sartorius Stedim, Goettingen, Germany). All gases were introduced to the bioreactor via the DZFO (Perlemax Ltd., UK) and proprietary diffusers, with air supplied for initial aerobic growth and mixtures of nitrogen and air supplied for fermentative growth. For continuous culture the gas mixtures were adjusted to give a redox potential of approximately -280 mV, (determined with an Easyferm Plus VP pH/Rx 255 electrode) in the reactor at steady state. All reagents were of analytical grade and sourced from Sigma Aldrich, UK.

### Reactor configuration

**Fig. 1:**
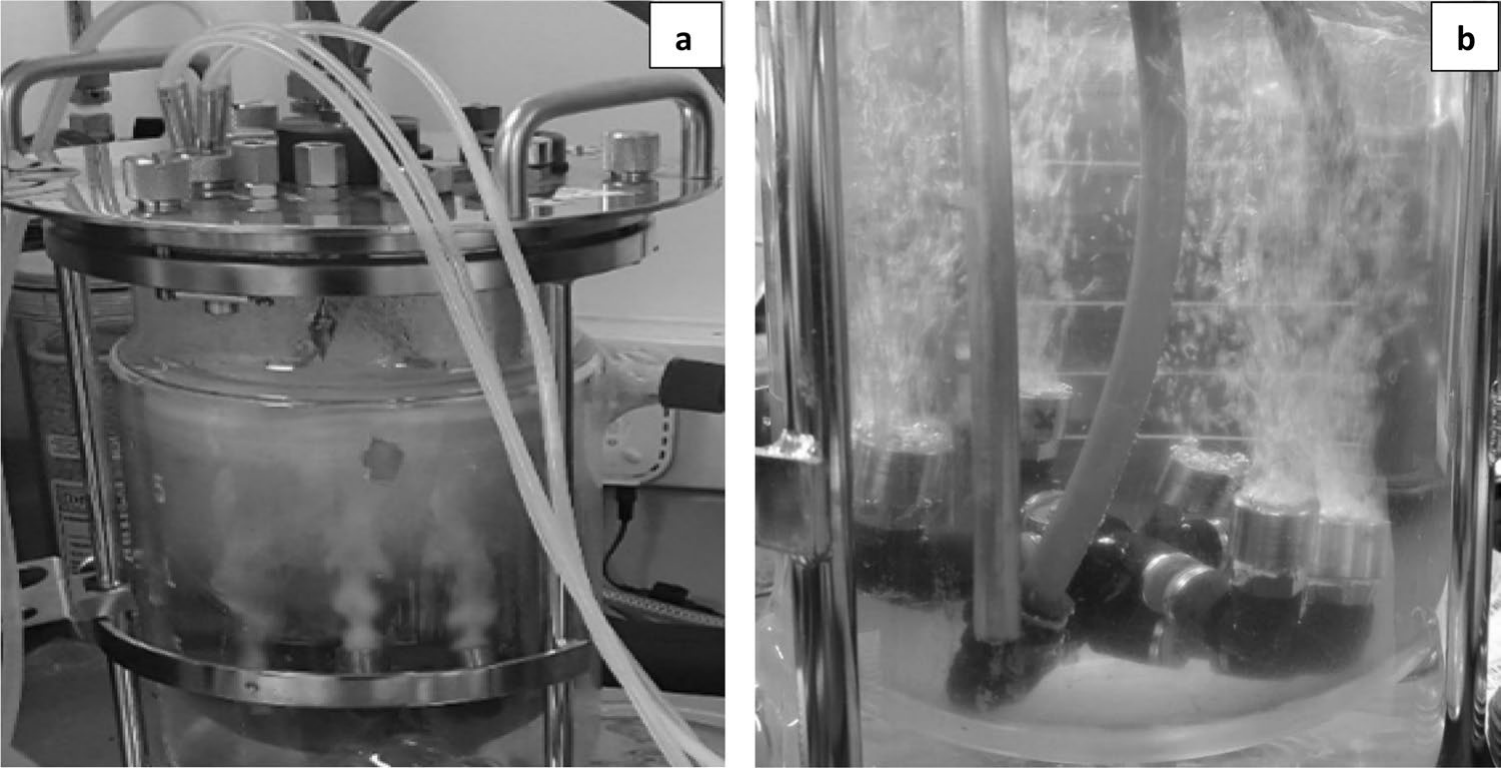
Bioreactor modified for microbubble stripping (**a**) Dense microbubble plume exiting the diffusers in fermentation broth (note the mushroom shape bubble plume, indicating oscillation of input gas) and (**b**) a close-up view of the diffuser assembly and gas inlet tubes in water

**Fig. 2:**
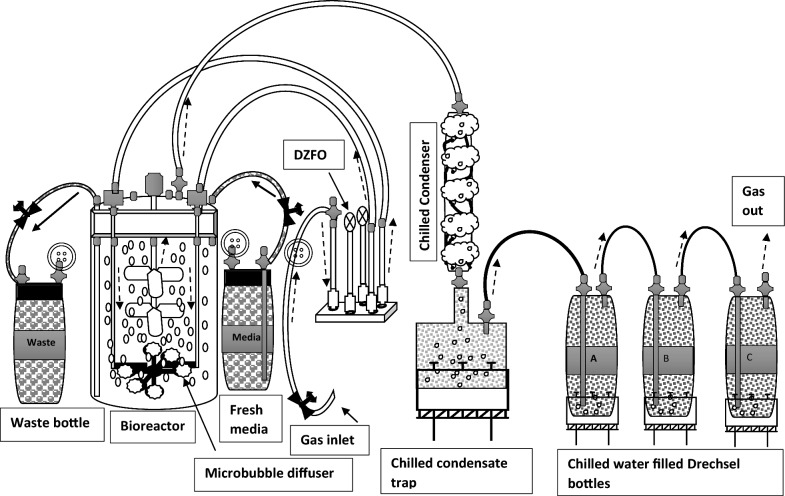
Schematic flow diagram of the continuous fermentation with fluidic oscillator (DZFO) generated microbubble aeration and ethanol in situ product extraction. Thick arrows show the direction of media or waste flow, while dashed arrows show the directional movement of gases.  = pump;  = ice bath;  = sterile filter;  = media flow;  = gas flow.

A Sartorius 2 L glass Univessel was modified to house a proprietary diffuser unit comprised of 6 diffuser outlets set in a ring (Figs. 1 and 2) and linked to a Desai-Zimmerman fluidic oscillator (self-excited fluidic binary switching device [33]) by 2 tubes secured in the headplate. For the fed batch experiment, the agitator shaft, sparger and baffles were removed to fit the diffuser into the vessel. Subsequently, for the continuous culture experiment, a shortened agitator shaft was fitted, with the bottom of the agitator shaft positioned approximately 2.5 cm above the diffusers. The Univessel condenser on top of the bioreactor was removed to minimise ethanol condensation and flow back into the reactor. This allowed ethanol vapours to escape from the reactor for subsequent collection in an external condenser and trap comprised of 2 glass condensers (QuickFit double walled) with internal coils in series, chilled with automobile antifreeze (Halfords, UK) from a Grant SS30 circulating bath cooler (Grant Instruments, UK), and a round-bottomed condensate trap placed in ice to collect the condensed ethanol vapours (see Fig. 2, only one glass condenser drawn, for clarity). Gases exiting from the condensate trap were then passed through a train of 3 chilled Drechsel bottles (A-C) containing chilled water, in order to trap any escaping ethanol. The collected ethanol in the condensate trap was removed at intervals and transferred to a collation bottle stored at 4 °C to minimize ethanol loss.

Unlike the configuration used in [34] the DZFO was designed to work with low gas flow rates, obviating the need for a gas vent; flow rates were controlled directly using the mass-flow controller in the Sartorius Biostat B control unit. A Sartorius 0.2 µm sterile filter was fitted between the control unit and the DZFO. The fundamental oscillation frequency was controlled by two resonant chambers—capped tubes of the same configuration as the inlet and outlet tubes, where the oscillation frequency was determined by the length of the resonant chamber (see Desai and Zimmerman [33]). The proprietary diffusers had a pore size distribution of 24–110 µm which were anisotropic in nature and had a hydrophilic surface. Unlike in previous studies, the gases were not heated before entering the reactor, primarily because the liquid height being used in the fed-batch experiment was variable and, in the continuous culture experiments, would have been sufficient to allow recondensation of the saturated vapours generated during initial contact when using hot microbubbles. The use of hot microbubbles is clearly a variable that can be explored in further experiments. The design and further information on this DZFO unit has been described previously [35], but in our case excluded the input heating system.

Because the diffuser and oscillator contained polypropylene parts they could not be autoclaved. Therefore, the bioreactor with all silicone tubing connections (media inlet and outlet tubes, all electrodes, gas outlet tube and gas delivery tube incorporating the filter were autoclaved separately at 121 ℃ for 15 min. All the other components, including the 2 gas inlet tubes and connectors between the DZFO and the diffusers, the DZFO itself, and diffuser array were sterilized by soaking in 70% ethanol for 24 h [36] and assembled in a class 2 cabinet after sterile air drying in the same cabinet.

### Bubble size measurement

Bubble size distributions were inferred using the laser diffraction approach, with the algorithm originally developed for particles and droplets by Swithenbank and coworkers [37] and commercialized by Malvern Instruments (UK), who adapted the approach to free streams of droplets with the Spraytec instrument. This was then expanded to work with bubbles and microbubble clouds by several others including Tesar [38] and Desai et al. [39].

Gas flowrates used in this study ranged between 0.52 vvm and 1.13 vvm, where vvm means the volumetric gas flowrate divided by the liquid volume in the reactor per minute. Software implementing the Swithenbank algorithm, bundled with the Spraytec, solving a well posed inversion of a time-averaged diffraction pattern from the scattered beam, was used to produce estimates of bubble size distribution. This was converted to size based on average number of bubbles. In all three cases, the bubble size estimates agreed well with a mean bubble size of 500 µm. It should be noted that the SprayTec approach does not detect smaller bubbles "hiding" behind larger bubbles [39]. It should also be noted that for small scale systems, smaller bubbles are not necessarily better due to the innately well mixed nature of the system as well as the potential for entrainment. For larger scale systems, this would be avoided and smaller bubbles would be of greater benefit. So bubble size was planned to be sufficient to prove the concept without incurring additional problems, although 100 fold smaller sizes have been achieved (cf. [35] and [30]) which provides a variable for subsequent experiments.

### Sample analysis

Samples (5 mL) were taken periodically, and the optical density at 600 nm (OD_600_), glucose and ethanol concentrations in the bioreactor, and ethanol concentration in the collation and the Drechsel bottles A-C were analysed and recorded. Volumes of ethanol collected in each bottle were also measured. Optical density was measured in a Jenway 6305 spectrophotometer using 3 mL cuvettes. All samples for HPLC analysis were immediately filtered through 0.2 µm nylon filters (Phenomenex Inc, Torrance, CA) and diluted as required (Raita et al. 2016) before storing at 4° C until analysis. Samples were separated by HPLC (Agilent 1200) on a 300 mm × 7.8 mm “organic acid” Rezex RHM Monosaccharide ROA H + column (Phenomenex Inc, Torrance, CA) with 5 mM H_2_SO_4_ acid as eluent running at 0.6 mL/min. The column was maintained at 65 ℃ in a G1316A column heater and 10 µL samples were injected automatically via a Rheodyne valve, with an analytical time of 25 min. Ethanol and residual glucose were detected and quantified by refractive index (G1362A), while acids were detected by UV at a wavelength of 215 nm (G1314B variable wavelength detector). Quantification was against standard curves of authentic > 99% purity reagents (Sigma Aldrich, UK) which were analysed after every 10 samples.

Maximum theoretical fermentative ethanol yields were calculated as 0.51 × glucose metabolised (g/g). For *P. thermoglucosidasius* TM242 measured yields are typically closer to 90% of theoretical [12], a yield level also typical of industrial yeast fermentations [40] so this value is also provided. Calculation of volumetric ethanol concentrations assumed that the density of ethanol at 25 ℃ is 0.785 g/mL.

## Results and discussion

### Continuously fed-batch and pulse fed-batch production of ethanol with gas stripping

**Fig. 3:**
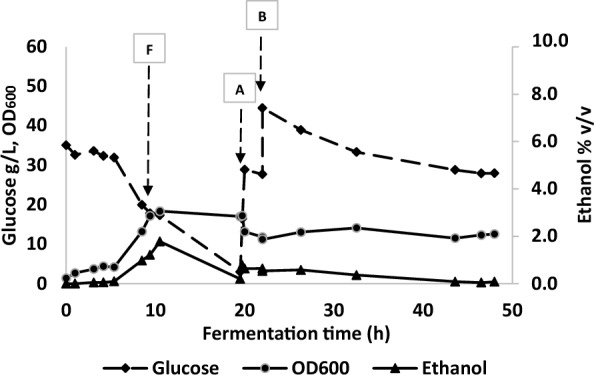
Fed-batch fermentation with in situ microbubble ethanol extraction without mechanical mixing. A low gas input of 0.52 vvm (15.3% oxygen, 84.7% nitrogen) was used. 2 batches (A, 150 mL and B 100 mL) of 250 g/L glucose media were added at 19.5 and 22 h. Arrow marked F indicates start of media feed

In a preliminary experiment to evaluate the performance of the bespoke microbubble diffuser, *P. thermoglucosidasius* strain TM242 was grown in fed-batch fermentation using the 2 L bioreactor vessel in which the stirrer shaft and impellers were completely removed to accommodate the microbubble diffusers (Fig. 1b). After 50 mL inoculation the starting volume was 750 mL and 1 vvm of air was supplied as microbubbles at room temperature for initial aerobic growth in 2 SPY medium containing 35 g/L glucose. After 3 h, when the OD_600_ had reached 3.7 in the starting growth medium, the gas supply was changed to a mixture of 15.3% (v/v) oxygen and 84.7% (v/v) nitrogen and passed through the diffuser at 0.52 vvm to initiate ethanol fermentation. After 9.75 h of operation, when the changes in glucose concentration, cell density, culture ethanol concentration (all in Fig. 3) and recovery (Additional file 1) and redox potential (Additional file 2) suggested that the cells were fully adapted to fermentative growth, filter-sterilized 2SPY media containing 250 g/L glucose was fed at 35 mL/h up to 19.5 h of operation. Following this, the continuous feed was switched off and a pulse of 150 mL, followed by a further 100 mL aliquot of the 250 g/L glucose feed solution was added after 19.5 h and 22 h of fermentation (Fig. 3).

By the end of the fermentation (48 h) approximately 600 mL of 250 g/L glucose feed media had been added, bringing the total liquid volume of the reactor to 1.13 L, after considering samples taken for analysis and volumes of liquid in the downstream traps, with a consequent reduction in gassing rate to 0.35 vvm. The initial liquid height from the bottom of the bioreactor was 9 cm, which had increased to approximately 13.6 cm by the end of the experiment, while the diffuser unit heights were 4, 6 and 8.5 cm (in pairs based on the joining arrangement). Figure 3 shows that over the initial batch and fed batch period (0–19.5 h) glucose was being used rapidly and, up to the point of the start of the fed batch, the cell density rose to an OD_600_ of nearly 20. Ethanol was clearly being produced after the switch to fermentative conditions and, despite the lack of significant increase in OD_600_ after 10 h (Fig. 3), ethanol production increased dramatically during the first phase of fed-batch, up to 19.5 h (Additional file 1). The period of fed batch growth from 9.75 to 19.5 h was overnight, during which the culture was not being monitored. As a result of the very low residual glucose encountered in the morning, two batches of fresh media were added. Unfortunately, glucose metabolism and ethanol production did not subsequently proceed at the same rate as previously observed, either because of metabolic stress encountered at the end of the first fed-batch phase, or possibly because the subsequent media addition pushed the glucose concentration close to the known [24] (nb in this work, mutants were obtained with much higher glucose tolerance) inhibitory concentration for this strain (40–50 g/L). Nevertheless, metabolism and ethanol production continued at a reduced rate (Fig. 3).

A further factor may have affected the rate of metabolism and ethanol production, which relates to the limitation of growth after approximately 10 h. Although described as a facultative anaerobe and clearly capable of producing fermentation products, it is evident that *P. thermoglucosidasius* requires a small amount of oxygen^1^ for growth under fermentative conditions when growing in a rich medium such as 2SPY. In our experience, growth and metabolic physiology are severely impacted at redox potentials of less than − 350 mV. Despite the beneficial effect of increasing surface area by using microbubbles, the lack of agitation in this experiment (which is typically the major driver of gas–liquid mass transfer in bioreactors) could have resulted in these cells becoming oxygen starved after 10 h, with a redox potential of − 400 mV at 10.5 h, dropping to − 440 mV after 19.7 h (Additional file 2). It should be noted that although the cell density barely changed over this fed batch period, the culture volume, and hence the total amount of cells increased by 47%. So the oxygen demand would have increased while supply remained constant.

Despite these issues, two significant findings are evident from this preliminary study. Firstly, that by calculating the total glucose usage and conversion to a theoretical ethanol concentration (based on previous studies using a low aeration rate in a rich medium [12] a yield of 90% of the theoretical maximum is achievable), this culture could have produced nearly 6% (v/v) ethanol (Table 1). Given that concentrations above 2% (v/v) are known to affect cell growth and growth completely stops at 4% (v/v), stripping using microbubbles has clearly enabled higher productivity. But perhaps more importantly, the ethanol concentration measured in the bioreactor throughout the 48 h period did not reach inhibitory levels. After 10.5 h, when the culture was in a rapid production phase (3.96 g/ L^−^h), the ethanol concentration was 1.78% (v/v). It is possible that it transiently rose to above 2% (v/v) overnight, but even if the metabolic rate had been reduced, the culture was clearly metabolically active and, by the time it was growing in the pure fed-batch phase the ethanol concentrations in the bioreactor had reduced. This was a good indication that gas stripping with microbubbles could allow a high ethanol productivity while maintaining sub-toxic concentrations in the liquid phase.

**Table 1:** Calculation of glucose consumed by *P. thermoglucosidasius* TM242 and predicted ethanol production during batch fermentation

*Glucose in 0.75/1.13 L (g)			**Ethanol yields (90%)		***Ethanol recovery in trapping bottles and condenser	
Total glucose	Glucose unused	Glucose consumed	ethanol g/L	ethanol v/v	Total g ethanol expected (corrected for sampling)	% Recovered in bottles (48 h)
173.0	31.6	141.4	56.2	7.2	63.5	61.4

*Total sugars in g were calculated by working out the concentrations of sugars in the reactor and volumes at the start (0.75 L) and end (1.13 L) of the process, including additions and reductions due to sampling and liquid stripped from the reactor into the collection vessels

**Yields were calculated based on the glucose used at a theoretical ethanol yield of 0.51 g/g glucose and assuming 90% conversion

*** Ethanol collected and analysed by HPLC

Unlike in the later experiments, only the condensate collation bottle was analysed during this preliminary experiment (Additional file 1), with the ethanol concentration and volumes in the Drechsel bottles simply being analysed at the end (results not shown). Based on the amounts recovered in all of the bottles, ethanol recovery, estimated over the entire 48h was only 61.4% of that expected (based on 90% of theoretical yield) which indicated that some losses were occurring. However, the calculated yield is based on total glucose utilisation, which includes the initial non-ethanologenic aerobic phase; additionally, it should be noted that some oxygen was going into the ethanol-producing batch and fed-batch culture, which would also reduce ethanol yields. Nevertheless, based on the measured recovery and the final volume in the bioreactor this would still imply production of a nominal 4.4% (v/v), which exceeds the toxic limit.

Direct measurement of instantaneous ethanol removal rates from the bioreactor is difficult because of rapidly changing productivities due to cell growth, changes in the bioreactor liquid volume, ethanol losses from the condenser unit/vapour traps and low productivities during different process stages. The highest rate of glucose utilisation and ethanol production (Additional file 1) corresponded to the fed batch phase between 9.75 h and 19.5 h. Although this period coincided with a fall in stripping efficiency due to the increase in culture volume (and consequent reduction in vvm), the dissolved ethanol levels were low at 19.5 h, suggesting that stripping rates were still adequate, and the culture glucose concentration was sufficiently high to indicate that productivity was not limited by the glucose feed rate. Although there would have been a linear increase in culture productivity due to the 47% increase in culture volume at constant cell density, the errors associated with averaging are significantly less than dealing with an exponentially growing culture (e.g. between 5.4 h and 9.4 h the cell density increased approximately fourfold.) Productivity during the final batch stages (2 and 3) was also easier to calculate as the cell density remained fairly constant. For these calculations (Table 2), it was assumed that 90% of the sugars consumed are converted to ethanol for maximum productivity comparison purposes [12].

**Table 2:** Ethanol production and removal rates for fed-batch and final batch mode

Stage	Time period (h)	Ethanol production rate (g/h)	Productivity (g/ L. h)	Removal rate by microbubbles (g/h)
1	9.4–19.5	4.31	3.96	5.33 (between 10.5–19.5)
2	20–22	0.34	0.28	0.29
3	22–48	0.39	0.29	0.57

The average ethanol productivity in fed-batch mode was found to be 3.96 g/L.h while ethanol was removed from the broth at the rate of 5.33 g/h by the microbubbles (2.5 g/L.h and 3.36 g/h based on measured ethanol recovery.) The gradual reduction in residual ethanol concentration in the bioreactor for this time period shown in Fig. 3 also confirms that the ethanol stripping rate was adequate, and exceeded the ethanol generation rate during this stage. The apparent increase in stripping rate between 10.5 h and 19.75 h (cf 5.4–9.75 h) may reflect the effects of media addition and an increase in volume {which increases bubble contact time) on a poorly mixed system. Calverley et al. [41] demonstrated that ethanol can be removed from a simulated bioreactor, producing the equivalent of a 10% (v/v) ethanol–water mixture, at a rate of 21.2 g/h with hot microbubbles (120 °C) while maintaining 1.23% (v/v) ethanol in the reactor. However, the maximum stripping rate reported in this study is only 25% of that reported by Calverley et al., [41] partly because the ethanol concentration in the reactor was not sufficient to maintain a high driving force and also because the feed gas used for stripping was supplied at a lower temperature (20 °C). Furthermore, biosurfactants present in the fermentation broth could also affect the stripping rate.

### Continuous culture with improved aeration to assess the limits of microbubble extraction

**Fig. 4:**
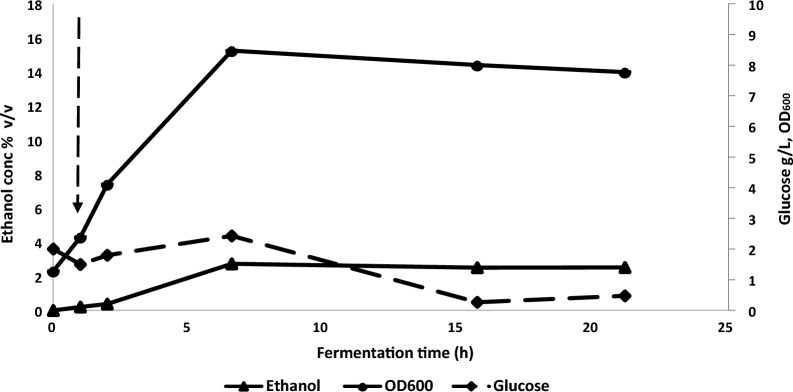
Cell (OD_600_), ethanol and residual glucose concentrations in the bioreactor during continuous culture (D = 0.1 /h) of *P. thermoglucosidasisus* TM242 at 60 °C with a medium feed of 35 g/L glucose and 7.7% (v/v) ethanol with in situ microbubble ethanol extraction at a total gas input of 1.13 vvm (O:N ratio = 0.126). Dashed arrow indicates start of media feed

The preliminary fed-batch fermentation demonstrated that gas-stripping of ethanol using microbubbles supplied at a moderate (for a lab scale bioreactor) volumetric flow rate was an effective way to keep ethanol concentrations below toxic levels. However, the specific productivity of the system was relatively low, and problems were encountered which were consistent with poor oxygen transfer into the culture. Given that the microbubbles served to both provide oxygen and strip out the ethanol, it was necessary to reintroduce an agitation system to provide some independent control over the former. This would then enable us to test the limits of microbubble stripping at realistic aeration rates [25]. To achieve this, a shortened (2/3 length) agitator shaft with 2 Rushton turbine impellers with approximately 2.5 cm spacing between the top of the diffuser unit and the bottom impeller and 2.5 cm spacing between impellers, was introduced into the bioreactor. Two additional modifications were made to allow the limits of microbubble extraction to be tested (under our operating conditions). Firstly, given the relative intolerance of *P. thermoglucosidasius* TM242 to high glucose concentrations [24], instead of providing high glucose concentrations in the feed, we mixed 35 g/L glucose and 7.7% (v/v) ethanol in 2SPY medium (to give a total of 9.75% (v/v) ethanol at steady state if all sugars were fermented) as shown in Table 3. While it is known that the effects of extracellular ethanol can differ from those of endogenously produced ethanol [42], the combination of the two should emulate those of produced ethanol, as high extracellular concentrations should restrict ethanol export, with consequent feedback effects on the ethanol production pathway. Additionally, the second set of experiments employed continuous culture with a 1.5 L fixed liquid volume in the bioreactor. This was necessary, primarily to provide the space to operate the agitator, but also meant that mass transfer efficiency would not vary with the liquid volume in the reactor. After some optimisation to ensure good stripping combined with adequate oxygen supply for fermentative growth, the gas input rates during continuous culture were set at 53% (v/v) air plus 47% (v/v) nitrogen (1.13 vvm total, O:N ratio = 0.126) with agitation at 100 rpm. The culture was started aerobically in batch (2SPY + 2g/L glucose) with a 5% (v/v) inoculum then, after 1h, switched to continuous culture using the medium supplemented with ethanol at a dilution rate of 0.1/h and the aeration regime described above. The gradual increase in ethanol concentrations in the culture, afforded by this regime, combined with regular monitoring of cell density, enabled assessment of the stage at which the stripping capacity became insufficient. However, a low concentration (1.8 g/L) of lactate appeared in the culture after 20 h of continuous operation, presumably as a result of contamination (as pointed out in methods, we were unable to autoclave the sparger unit.) Feasibly, this could have been due to reversal of the lactate utilization (Lut) pathway, although this was not observed in the preliminary experiment. At this point the culture ethanol concentration should have been approx. 9.75% (v/v) based on a residual glucose concentration of 0.5 g/L and assuming 90% of theoretical ethanol production (not accounting for losses due to lactate production). It should be noted that there was no significant reduction in ethanol concentration associated with the initial appearance of lactate, but the residual glucose concentration dropped. After 20 h of continuous feeding the measured ethanol concentration in the culture was approximately 2.5% (v/v); sufficient to cause some effect on growth rate but not complete inhibition (Fig. 4).

**Table 3:** Ethanol yields predicted from continuous culture experiment with ethanol supplementation to the broth

Source of data	Glucose g/L	*Ethanol yields (90% of theoretical)		Ethanol recovery in condenser and trapping bottles	
		Ethanol g/L	Ethanol v/v	Predicted ethanol (g)**	% of expected ethanol recovered in bottles and the condenser
Sugar in feed	35	16.07	2.05	176.7	70.1 (***74.2)
Ethanol in feed		60.45	7.70		
Total	35	*76.52	9.75		

*Expected ethanol yields were calculated based on the sugar feed at a theoretical yield of 90% of 0.51 g ethanol per g of glucose, plus ethanol already in the feed media. ** Predicted amount of ethanol stripped with microbubbles, based on that generated and added (76.52 g/L at D = 0.1 /h) minus the amount exiting the bioreactor via the overflow and through sampling. *** Ethanol recovery between 6.2 and 21.17 h. Expected yield ignores the glucose present in the bioreactor at t = 1 h and at the end of the experiment, and the unmetabolised glucose that exited via the overflow

From experimental measurement of the changes in concentration and volume in the condensate trap and Drechsel bottles (Additional files 3 & 4) the average ethanol stripping rate recorded between 6.2 and 21.2 h of continuous culture, was 6.43 g/h. After 6h the concentration in the condensate trap stabilised at approximately 30% (v/v), while the volume and concentration of ethanol in all of the Drechsel bottles steadily increased throughout the run (Additional file 7). After 20 h of continuous operation the ethanol concentration in Drechsel bottle A was 17.5% (v/v), while bottle B and C were 8.3% and 1.5% respectively (Additional file 3).

Although this culture did not reach steady state, it was clear that, with a nominal production of approximately 9.75% (v/v) ethanol and redox at − 218 mV (Additional file 4), the operating conditions being used were not able to keep the ethanol concentration in the culture below 2% (v/v). However, by running a continuous culture at a dilution rate below the potential maximum growth rate of TM242, a marginal reduction in maximum growth rate caused by ethanol inhibition would not have been evident (although this may have contributed to the presence of residual glucose in the culture).

It should be noted that even with the initial aerobic phase excluded and consistent growth with a low redox potential throughout the continuous culture, recovered ethanol still only comprised 70–74% of predicted production (and was marginally less than the added ethanol) which suggests that most of the losses arise from incomplete recovery. In our previous work using ethanol–water mixtures with a similar recovery train as employed here [41], ethanol recovery was consistently less than 60%, and Schlafle et al. [43] have reported similar problems. From an experimental perspective, direct measurement of ethanol leaving the reactor in the vapour phase would be preferable. However, particularly as these cultures were maintained at low redox potential, it is reasonable to conclude that the actual ethanol productivities are much closer to the theoretical values based on glucose usage than the experimental results suggest.

To analyse the productivity of the cells and the removal rate by microbubbles for continuous culture, it is necessary to establish a mass balance over the system.

Mass balance for sugars

1 $$\frac{d}{dt}\left({V}_{b}{C}_{s}\right)=F{C}_{F,S}-F{C}_{S}-{U}_{S}$$

where ${V}_{b}$ is the bioreactor volume, ${C}_{s}$ is the instantaneous sugar concentration in the bioreactor,${C}_{F,S}$ is the sugar concentration in feed, $F$ is the liquid flow rate in and out of the bioreactor, ${U}_{S}$ is the sugar consumption rate and $t$ is the time.

Mass balance for ethanol

2 $$\frac{d}{dt}\left({V}_{b}{C}_{E}\right)=F{C}_{F,E}-F{C}_{E}+{G}_{E}-R$$

${C}_{E}$ is the instantaneous ethanol concentration in the bioreactor, ${C}_{F,E}$ is the ethanol concentration in the feed, ${G}_{E}$ is the ethanol generation rate by microbes and $R$ is the ethanol removal rate by microbubbles.

Based on 90% of theoretical yield,

3 $${U}_{s}\times 0.51\times 0.9={G}_{E}$$

Assuming linear changes in concentration profiles shown in Fig. 4, average production and removal rates can be estimated using Eqs. (1) to (3). Results are shown in Table 4.

**Table 4:** Ethanol production and removal rates for continuous mode

Time period (h)	Ethanol production rate, ${G}_{E}$ (g/h)	Productivity (g L^−1^ h^−1^)	Removal rate by microbubbles, $R$(g/h)	Ethanol concentration range in bioreactor (g/L)
2–6.6	2.1	1.4	2.0	3.0–19.8
6.6–15.7	2.6	1.7	9.0	19.8–19.7
15.7–21.2	2.4	1.6	8.4	19.7–19.9

Given that the measured ethanol recovery between 6.6 and 21.2h was only 74% of that predicted, then the productivity and removal rate figures would need to be multiplied by 0.74 to generate measured values. When comparing ethanol stripping rates reported for the fed-batch mode operation (Table 2) with those for the continuous operation (Table 4), it is evident that for most time periods, the microbubble assisted stripping rate was higher during continuous operation. However, it appears that this stripping rate was insufficient to keep the residual ethanol concentration in the bioreactor below 2% (v/v) at the high production rates (actually production plus supplementation rates) achieved in this experiment.

### Chemostat culture with microbubble-assisted ethanol removal

**Fig. 5:**
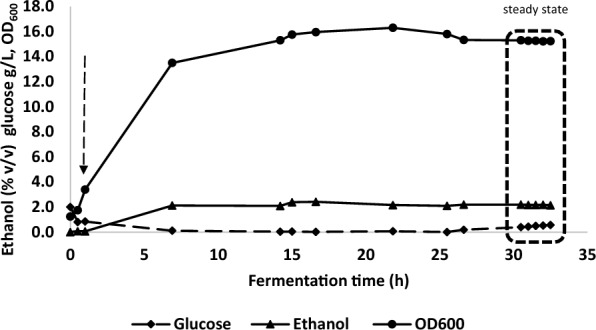
Continuous culture fermentation of TM242 at D = 0.1 /h, redox − 280 mV (Additional file 5) with a feed of 35 g/L glucose + 5% v/v ethanol and in situ microbubble stripping at 1.13 vvm (at an O:N ratio of 0.126.) Dashed arrow indicates start of continuous media feed

**Fig. 6:**
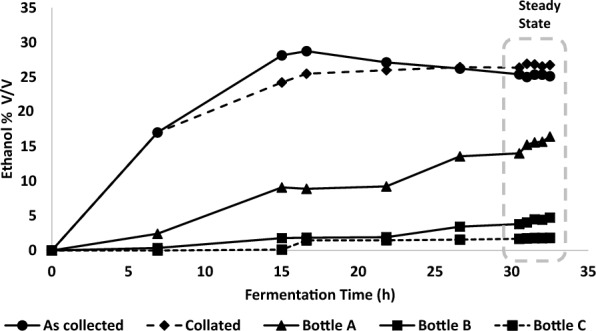
Composition of bioreactor exhaust vapour condensate samples taken at different time points (“as collected”) from the condenser trap, collated samples of this condensate, and Drechsel bottles A to C

It was evident from the continuous culture study above that the operating conditions were pushing the limits of the current system. While it may have been possible to optimise the operating conditions further to have achieved steady state operation with close to 10% (v/v) ethanol, the primary purpose of this work was to demonstrate that efficient gas stripping of ethanol using microbubbles would allow continuous operation under realistic operating conditions. Therefore, as a pragmatic next step, the nominal ethanol concentration was reduced to approximately 7% (v/v) in order to run the process as a carbon limited chemostat without ethanol inhibition. As before, the fermentation medium was supplemented with ethanol, in this case 5% (v/v); together with fermentation of 35 g/L glucose this should produce a nominal 7.0–7.2% (v/v) final ethanol concentration based on previous assumptions (Table 5). As in the continuous culture run, cells were grown aerobically in batch culture (2SPY + 2 g/L glucose) before switching to continuous culture at D = 0.1 /h using the same aeration and agitation conditions as previously described. Unlike in the previous run, residual glucose concentrations fell to near zero for the majority of the run, while ethanol concentrations and redox in the culture stabilised at around 2.17% (v/v) and − 238 mV respectively (Fig. 5 and Additional file 5). The ethanol concentration was lower than observed in the previous run and any toxicity effects were not sufficient for the culture to be restricted from reaching carbon limitation. As noted in the initial continuous culture run, the introduction of ethanol into the feed would have resulted in a gradual increase in nominal ethanol concentration in the bioreactor. After 30 h of continuous culture the residual glucose concentration, cell concentration and bioreactor ethanol concentration had been stable for the previous 15h and were considered to be in gross physiological steady state. (Despite many years of use, there is no consistent definition of the number of dilutions/generations required to reach steady state, and it may vary depending on the type of questions being asked [44, 45]. Detailed systems biology studies of cellular components may require more generations to reach stability, but during this time cells are under constant evolutionary pressure [46].) The fact that the culture appeared to be glucose limited and the bioreactor ethanol concentration was close to 2% (v/v) was convincing evidence that a chemostat producing a nominal 7% (v/v) operating at D = 0.1 /h is feasible using the current gas-stripping configuration. In this instance, no lactate production was observed. After 15–16 h of continuous culture, the ethanol concentration of the sequentially collected samples from the condenser was 26.4–26.9% v/v, while the final concentration of ethanol in the collation bottle (total 340 mL) was 26.7% v/v (Fig. 6). Over the course of the chemostat culture the ethanol trapping bottles A, B and C had increased in volume by 39 mL, 10 mL and 10.5 mL respectively (Additional file 6), with final ethanol concentrations of 16.4, 7.4 and 1.8% v/v respectively (Fig. 6). This gave a measured ethanol recovery of 76% of the theoretical level of production over the whole experiment, or 72% using the steady state data from the last 10 h of operation (results not shown). The presence of ethanol in bottle C again suggested that some ethanol was being lost from the system. The measured ethanol stripping rate between 20 and 30 h of the chemostat culture was 4.0 g/h.

**Table 5:** Calculated total ethanol from continuous steady state fermentation simulated with both glucose and ethanol in the broth

Source of data	Sugars g/L	*Ethanol yields (90% of theoretical)		Ethanol recovery in trapping bottles	
		Ethanol g/L	Ethanol % (v/v)	**Predicted ethanol (g)	% of predicted ethanol recovered
Sugar in feed	35	16.07	2.05	143.9	76.0
Ethanol in feed		39.25	5.00		
Total	35	55.32	7.05		

*Expected nominal ethanol concentrations were calculated based on the sugar feed at 90% of a theoretical maximum yield of 0.51 g ethanol per g glucose, plus ethanol already in the feed media. ** Predicted ethanol stripping is based on the theoretical production levels minus ethanol removed in the overflow and lost through sampling

In this experiment, quasi-steady-state is achieved during the time period 14–26 h, and the bioreactor performance can be analysed using the mathematical model presented earlier (Eqs. 1–3) for continuous culture with improved aeration. In this case, the accumulation terms in Eqs. (1) and (2) are zero, and ${G}_{E}$ and $R$ can be found by solving Eqs. (1), (3) and (2), sequentially. The ethanol generation rate by the microbes, productivity and ethanol removal rate by microbubbles is calculated as 2.4 g/h, 1.6 g/L.h and 5.7 g/h respectively (multiply these figures by 0.76 to give values based on measured data). Even though the ethanol concentration remained at 2.09–2.2% (v/v) in this case, providing a high driving force for mass transfer compared to the cases considered by Calverley et al. [32, 34], the stripping rate was much lower, primarily because the microbubbles were generated at room temperature. This could be alleviated by pre-heating the gas stream used for microbubble stripping, and operating the stripping process either *in-situ* or using an external microbubble stripping unit where recondensation back into the bulk liquid can be minimised [32].

Overall, these experiments demonstrate the potential of in situ microbubble stripping for continuous ethanol removal from a bioreactor. This stripping rate would not have been achieved using sparger aeration as a very low rate of agitation was used, which would have had very little effect on the size of sparger generated bubbles [25]. In order to increase stripping rates, to reduce the residual ethanol concentration below 2% (v/v), further refinements are necessary. Maintaining a heated flow path from the bioreactor to the condenser would lead to some improvement, by minimising recondensation into the bioreactor, while increasing the overall diffuser area, still maintaining a low gas flux through the diffuser should also lead to improvements (this is much simpler at large scale than in lab scale bioreactors). However, by far the most important variable lies in the microbubble properties. Much smaller bubbles can be produced with the DZFO, which would dramatically increase the interfacial surface area per unit volume, increasing the stripping efficiency per unit gas volume, while heating the gas to 90–150 °C significantly increases the ethanol stripping rate [34]. Strong internal mixing [28] and high gas temperatures within microbubbles facilitates high absolute vapour content in bubbles (both ethanol and water) for a short time; but gradual condensation and sensible heat transfer to the liquid will reduce the absolute vapour content if the bubbles are retained in the liquid for a longer period. Hence, in general, higher gas temperatures and lower liquid heights are preferred for improved stripping. According to Calverley et al. [34], raising the gas temperature from 90 °C to 120 °C caused an increased stripping rate for liquid layer heights of 5–25 mm but did not produce a noticeable advantage for 50 mm, unless the gas temperature was increased to 150 °C. In light of this result, a much higher gas temperature (exceeding 150 °C) would be required in these *in-situ* experiments to achieve an advantage over low gas temperature stripping, which used a liquid layer height of ~ 120 mm (distance from the bubbling membrane to free liquid surface). Since an agitation system was introduced to the bioreactor following fed-batch operation, it may not be possible to reduce this liquid height. However, a side arm microbubble distillation unit, that operates in a continuous flow through fashion at liquid heights less than 10 cm would result in substantially better removal rates and could be operated at lower temperatures [32].

## Conclusions

The Desai-Zimmerman fluidic oscillator provides an energy efficient method to generate microbubbles of controllable size [47]. Although it has been shown to efficiently strip ethanol from pure ethanol–water mixtures, this is the first time that its intrinsic value for in situ stripping of ethanol from thermophilic fermentations has been demonstrated. Given that the growth temperature of these organisms is close to the boiling point of ethanol the concept of continuous gas stripping or vacuum stripping has been recognised for some time. However, unless unrealistically high gassing rates are used, stripping using standard spargers is too slow to be useful [25], primarily because sparger-generated bubbles are relatively large thereby producing too little surface area for mass transfer and inefficient internal mixing compared with DZFO generated microbubbles. The generation of microbubbles increases the gas–liquid contact area for the same volume of gas, thus directly enhancing mass transfer of ethanol into the gas phase (gas transfer rate α k_L_a, where a is the interface surface area per unit volume).

Using a bespoke diffuser, specifically designed to work in a laboratory 1.5 L bioreactor, we have demonstrated that, without extensive optimisation, it was possible to operate a chemostat culture of *P thermoglucosidasius* TM242 at a nominal ethanol concentration of 7% (v/v), using microbubbles passing through the culture at 1.13 vvm to maintain the solution ethanol concentration at around 2% (v/v). Complete condensation of the resulting vapour stream yielded a concentrated (> 25% v/v) ethanol solution, but this could have been increased by employing fractional distillation, thus significantly reducing the costs of final distillation. Although the operation also produces a low concentration fermentation broth, hot microbubble stripping of the latter should allow recovery of much of this fraction, and the resulting condensate can be combined with the first for final distillation.

While continuous production of 7% (v/v) ethanol would already represent commercially viable levels, particularly as steady state operation allows continuous rather than batch operation, there is plenty of room for improvement. The design of the system required the use of relatively large microbubbles due to small size of the reactor. Indeed, bubbles this size could potentially have been generated using a traditional sparger combined with high agitation rates [48]. However, with a scaled up system, stripping can be achieved using smaller microbubbles and low agitation rates, as observed by Desai et al. [30] for in situ ammonia removal. Use of 100 μm microbubbles [30] at 40% of the gas flow rate used in the current study would double the interfacial area for gas stripping, but taking account of the increased gas hold up of smaller bubbles the specific interfacial area would be much higher. Therefore, commercially useful stripping rates should be achievable using this approach, even with microbubbles generated at room temperature. Alternatively, as previously demonstrated, reducing contacting time by using a recirculating loop would allow the use of higher gas temperatures, thus increasing the efficiency of separation.

Finally, even a marginal increase in ethanol tolerance of the host organism, which has recently been achieved [24], would allow the system to operate at both higher actual and nominal ethanol concentrations.

## Supplementary Information

Additional file 1. Liquid volume and concentration of ethanol in the condensate collation bottle in the non-agitated continuously and pulse fed-batch fermentation with in situ microbubble ethanol extraction.

Additional file 2. Redox potential readings for continuously and pulse fed-batch fermentation without mechanical stirring.

Additional file 3. Concentration of ethanol in the collation bottle and downstream ethanol trap bottles A-C in continuous culture experiment with mechanical stirring. Bottles A, B and C were used to trap ethanol escaping the main collection bottle and contained 200, 100 and 200mL of water at time zero, respectively. The bioreactor media feed was at a dilution rate of 0.1/h.

Additional file 4. Volumes of aqueous ethanol recovered in downstream collection vessels during continuous culture using mechanical stirring, with* in situ* microbubble extraction. At time zero Drechsel bottles A, B and C, downstream of the condensate collector contained 200mL, 100mL and 200mL water respectively to trap ethanol escaping from the condensate colllector. The collation bottle contained the total volume of condensate collected and decanted at intervals during the experiment.

Additional file 5. Redox potential during continuous culture with mechanical stirring and in situ microbubble extraction.

Additional file 6. Redox potential during chemostat fermentation.

Additional file 7. Volumes of aqueous ethanol recovered in downstream collection vessels during chemostat culture with in situ microbubble extraction. At time zero Drechsel bottles A, B and C, downstream of the condensate collector contained 200 mL, 100 mL and 200 mL water respectively to trap ethanol escaping from the condensate collector. The collation bottle contained the total volume of condensate collected and decanted at intervals during the experiment.

## Data Availability

No datasets were generated or analysed during the current study.

## Abbreviations

$${V}_{b}$$: Bioreactor volume, L
$$t$$: Time, h
${C}_{s}$: Instantaneous sugar concentration in the bioreactor, g/L
${C}_{E}$: Instantaneous ethanol concentration in the bioreactor, g/L
${C}_{F,S}$: Sugar concentation in feed, g/L
${C}_{F,E}$: Ethanol concentation in feed, g/L
$F$: Liquid flow rate in and out of the bioreactor, L/h
${U}_{S}$: Sugar consumption rate, g/h
${G}_{E}$: Ethanol generation rate by microbes, g/h
$R$: Ethanol removal rate by microbubbles, g/h

## Footnotes

1 Work done subsequently has identified a missing medium component which allows *P. thermoglucosidasius* to grow fully anaerobically.
