# Supplementary material for: Toxicity reduction in continuous, high productivity ethanol fermentation by Parageobacillus thermoglucosidasius using in situ microbubble gas stripping
Source: Microb Cell Fact. 2025 Jun 18;24:137. doi: 10.1186/s12934-025-02754-5 (PMC12177972; doi:10.1186/s12934-025-02754-5)
Supplement: Supplementary file 1 — Additional file 1. Liquid volume and concentration of ethanol in the condensate collation bottle in the non-agitated continuously and pulse fed-batch fermentation with in situ microbubble ethanol extraction. [file 12934_2025_2754_MOESM1_ESM.pdf]

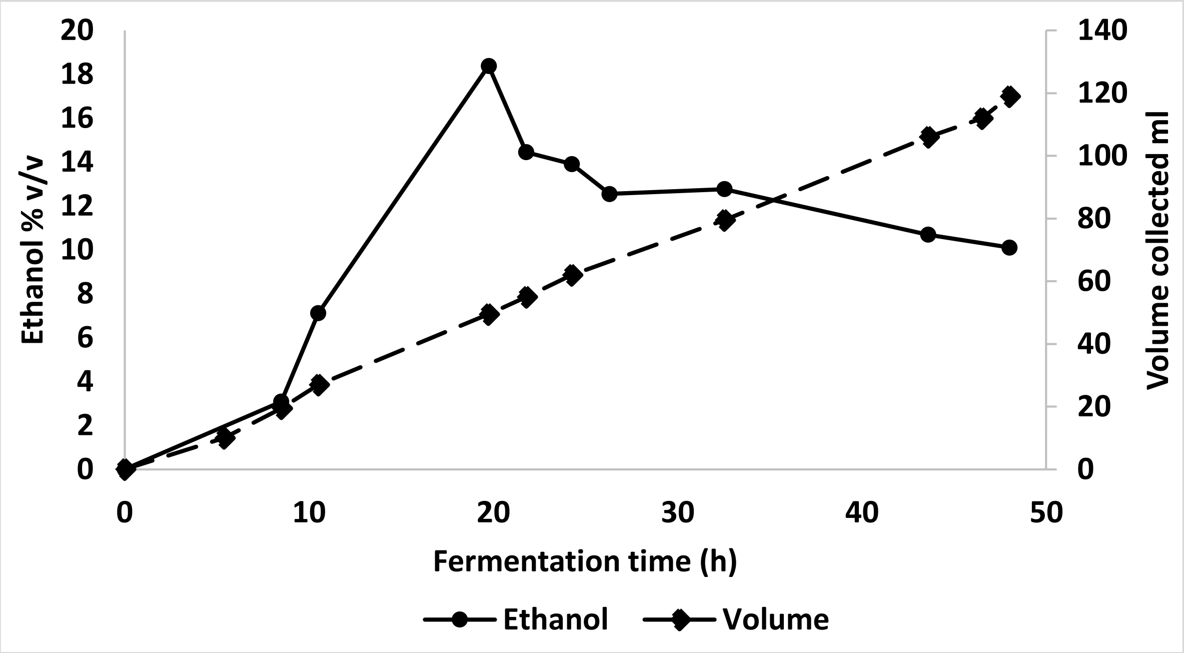

Additional file 1. Liquid volume and concentration of ethanol in the condensate collation bottle in the non-agitated continuously and pulse fed-batch fermentation with *in situ* microbubble ethanol extraction.
