# Supplementary material for: Toxicity reduction in continuous, high productivity ethanol fermentation by Parageobacillus thermoglucosidasius using in situ microbubble gas stripping
Source: Microb Cell Fact. 2025 Jun 18;24:137. doi: 10.1186/s12934-025-02754-5 (PMC12177972; doi:10.1186/s12934-025-02754-5)
Supplement: Supplementary file 2 — Additional file 2. Redox potential readings for continuously and pulse fed-batch fermentation without mechanical stirring. [file 12934_2025_2754_MOESM2_ESM.pdf]

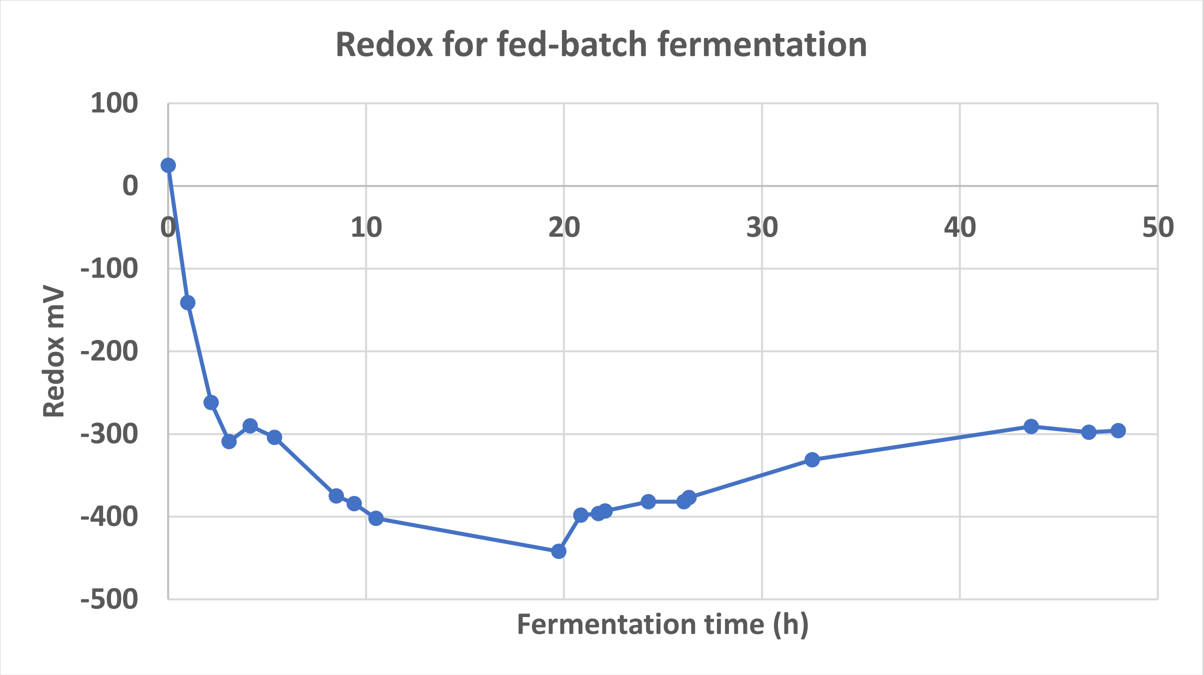

Additional File 2: Redox potential readings for continuously and pulse fed-batch fermentation without mechanical stirring.
