# Supplementary material for: Toxicity reduction in continuous, high productivity ethanol fermentation by Parageobacillus thermoglucosidasius using in situ microbubble gas stripping
Source: Microb Cell Fact. 2025 Jun 18;24:137. doi: 10.1186/s12934-025-02754-5 (PMC12177972; doi:10.1186/s12934-025-02754-5)
Supplement: Supplementary file 3 — Additional file 3. Concentration of ethanol in the collation bottle and downstream ethanol trap bottles A-C in continuous culture experiment with mechanical stirring. Bottles A, B and C were used to trap ethanol escaping the main collection bottle and contained 200, 100 and 200mL of water at time zero, respectively. The bioreactor media feed was at a dilution rate of 0.1/h. [file 12934_2025_2754_MOESM3_ESM.pdf]

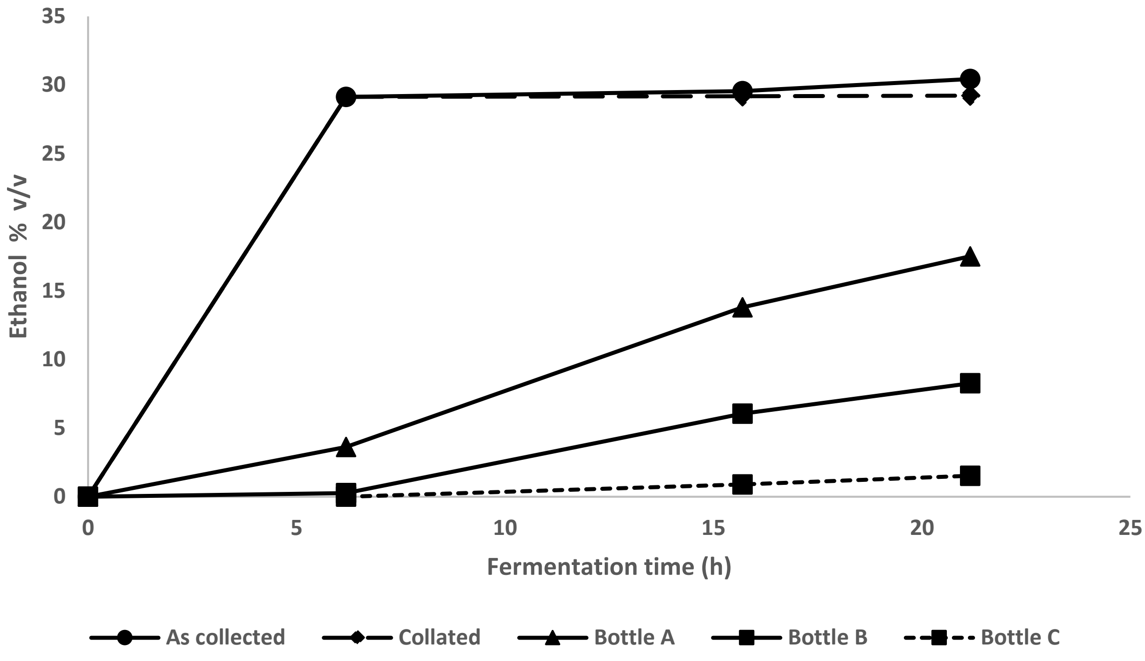

Additional file 3: Concentration of ethanol in the collation bottle and downstream ethanol trap bottles A-C in continuous culture experiment with mechanical stirring. Bottles A, B and C were used to trap ethanol escaping the main collection bottle and contained 200, 100 and 200mL of water at time zero, respectively. The bioreactor media feed was at a dilution rate of 0.1/h
