# Supplementary material for: Toxicity reduction in continuous, high productivity ethanol fermentation by Parageobacillus thermoglucosidasius using in situ microbubble gas stripping
Source: Microb Cell Fact. 2025 Jun 18;24:137. doi: 10.1186/s12934-025-02754-5 (PMC12177972; doi:10.1186/s12934-025-02754-5)
Supplement: Supplementary file 4 — Additional file 4. Volumes of aqueous ethanol recovered in downstream collection vessels during continuous culture using mechanical stirring, with in situ microbubble extraction. At time zero Drechsel bottles A, B and C, downstream of the condensate collector contained 200mL, 100mL and 200mL water respectively to trap ethanol escaping from the condensate colllector. The collation bottle contained the total volume of condensate collected and decanted at intervals during the experiment. [file 12934_2025_2754_MOESM4_ESM.pdf]

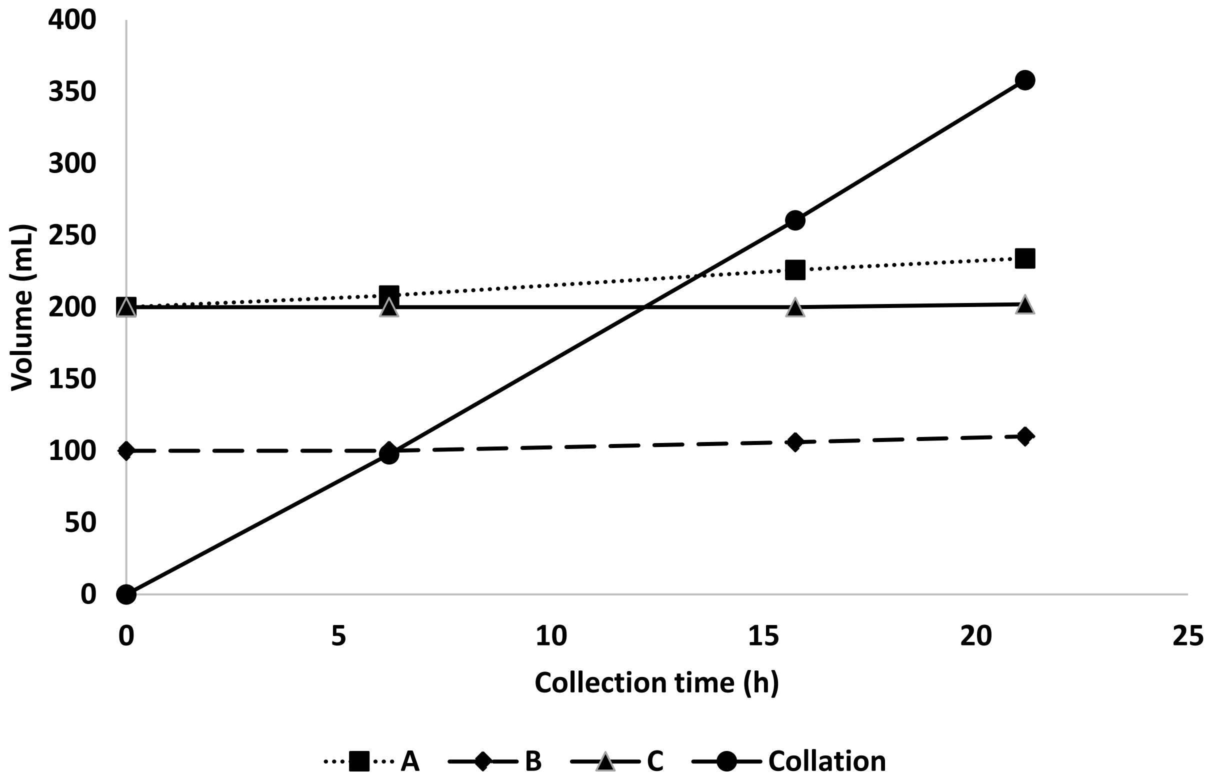

Additional file 4: Volumes of aqueous ethanol recovered in downstream collection vessels during continuous culture using mechanical stirring, with *in situ* microbubble extraction. At time zero Drechsel bottles A, B and C, downstream of the condensate collector contained 200mL, 100mL and 200mL water respectively to trap ethanol escaping from the condensate colllector. The collation bottle contained the total volume of condensate collected and decanted at intervals during the experiment.
