# Supplementary material for: Toxicity reduction in continuous, high productivity ethanol fermentation by Parageobacillus thermoglucosidasius using in situ microbubble gas stripping
Source: Microb Cell Fact. 2025 Jun 18;24:137. doi: 10.1186/s12934-025-02754-5 (PMC12177972; doi:10.1186/s12934-025-02754-5)
Supplement: Supplementary file 5 — Additional file 5. Redox potential during continuous culture with mechanical stirring and in situ microbubble extraction. [file 12934_2025_2754_MOESM5_ESM.pdf]

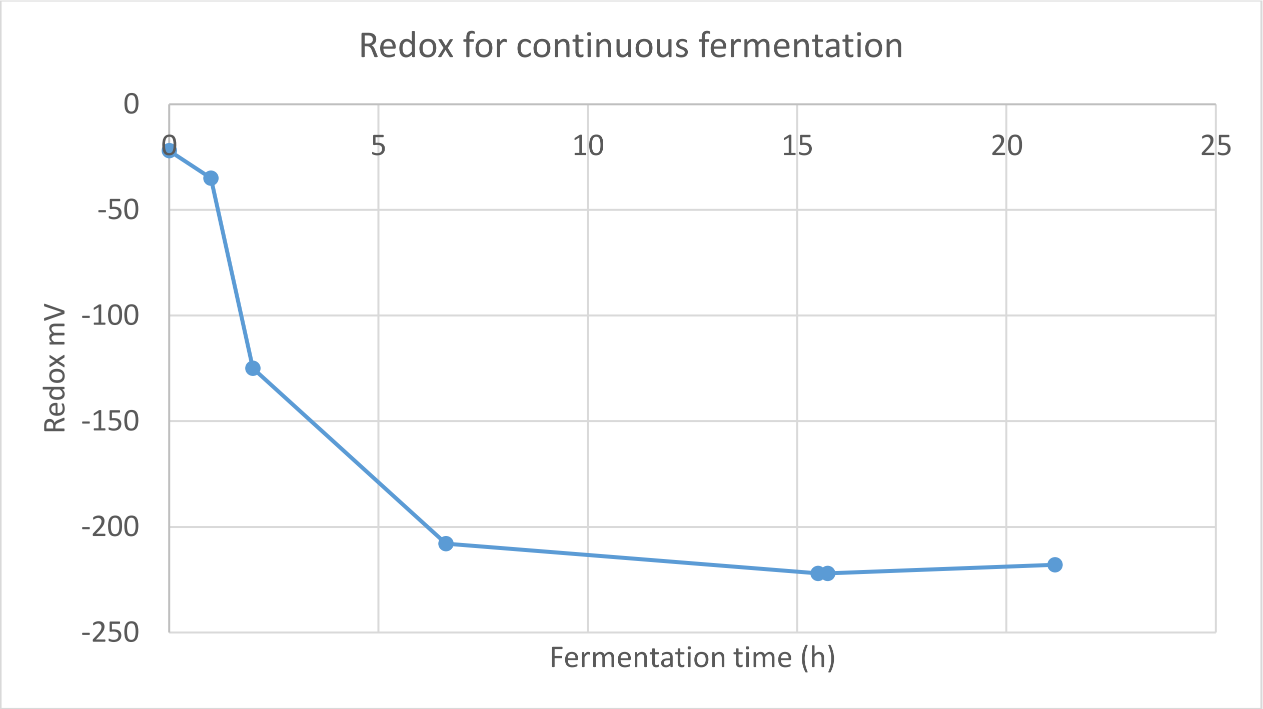

<u>Additional file 5</u>: Redox potential during continuous culture with mechanical stirring and *in situ* microbubble extraction.
