# Supplementary figures and images for: Toxicity reduction in continuous, high productivity ethanol fermentation by Parageobacillus thermoglucosidasius using in situ microbubble gas stripping
Source: Microb Cell Fact. 2025 Jun 18;24:137. doi: 10.1186/s12934-025-02754-5 (PMC12177972; doi:10.1186/s12934-025-02754-5)

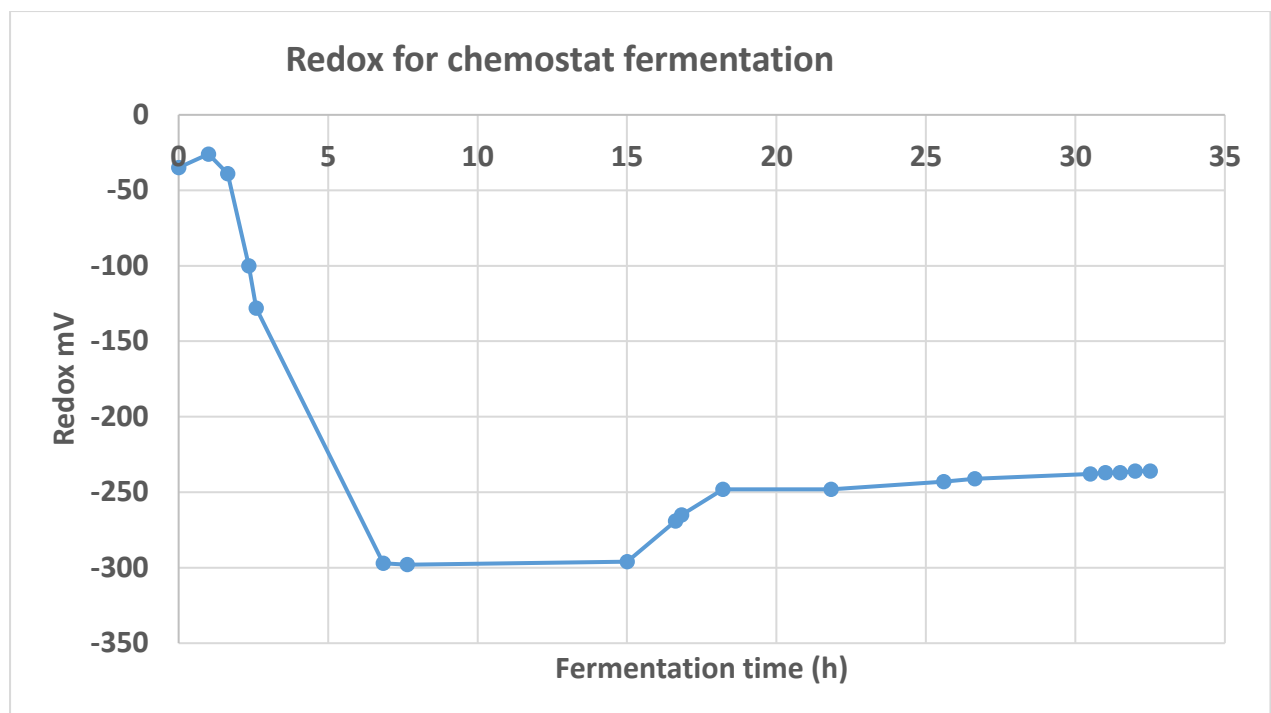

Additional file 6: Redox potential during chemostat fermentation.

Supplement: Supplementary file 6 — Additional file 6. Redox potential during chemostat fermentation. [file 12934_2025_2754_MOESM6_ESM.pdf]
